# Poultry diseases diagnostics models using deep learning

**DOI:** 10.3389/frai.2022.733345

**Published:** 2022-08-01

**Authors:** Dina Machuve, Ezinne Nwankwo, Neema Mduma, Jimmy Mbelwa

**Affiliations:** ^1^Department of IT Systems Development and Management, Nelson Mandela African Institution of Science and Technology, Arusha, Tanzania; ^2^Department of Electrical Engineering and Computer Science, University of California, Berkeley, Berkeley, CA, United States; ^3^Department of Computer Science and Engineering, University of Dar es Salaam, Dar es Salaam, Tanzania

**Keywords:** deep learning, agriculture, poultry disease diagnostics, dataset, image classification

## Abstract

Coccidiosis, Salmonella, and Newcastle are the common poultry diseases that curtail poultry production if they are not detected early. In Tanzania, these diseases are not detected early due to limited access to agricultural support services by poultry farmers. Deep learning techniques have the potential for early diagnosis of these poultry diseases. In this study, a deep Convolutional Neural Network (CNN) model was developed to diagnose poultry diseases by classifying healthy and unhealthy fecal images. Unhealthy fecal images may be symptomatic of Coccidiosis, Salmonella, and Newcastle diseases. We collected 1,255 laboratory-labeled fecal images and fecal samples used in Polymerase Chain Reaction diagnostics to annotate the laboratory-labeled fecal images. We took 6,812 poultry fecal photos using an Open Data Kit. Agricultural support experts annotated the farm-labeled fecal images. Then we used a baseline CNN model, VGG16, InceptionV3, MobileNetV2, and Xception models. We trained models using farm and laboratory-labeled fecal images and then fine-tuned them. The test set used farm-labeled images. The test accuracies results without fine-tuning were 83.06% for the baseline CNN, 85.85% for VGG16, 94.79% for InceptionV3, 87.46% for MobileNetV2, and 88.27% for Xception. Finetuning while freezing the batch normalization layer improved model accuracies, resulting in 95.01% for VGG16, 95.45% for InceptionV3, 98.02% for MobileNetV2, and 98.24% for Xception, with F1 scores for all classifiers above 75% in all four classes. Given the lighter weight of the trained MobileNetV2 and its better ability to generalize, we recommend deploying this model for the early detection of poultry diseases at the farm level.

## 1. Introduction

The continent of Africa contributes 10% to the global poultry population of 23 billion live chickens (FAO, 2013; FAOSTAT, 2016). The GDP contribution from the poultry sector in Tanzania was valued at 76 million USD in 2017 (Michael et al., 2018). Tanzania has the third largest livestock population in Africa with 36 million chickens in 4.6 million households (27 million people) (URT, 2015). However, there are many challenges that farmers in Tanzania face that have led to low productivity (URT, 2015). In particular, the poultry sector is challenged by low productivity due to diseases such as Salmonella, Newcastle and Coccidiosis. The economic effects of such widespread poultry diseases include high mortality rates and failure to compete on the export market with other high producing countries (FAO, 2013). In addition, there are other, more broad, downstream effects of farm and crop diseases that include food insecurity in specific regions and economic instability for small scale farmers (Michael et al., 2018). This makes the impact of poultry diseases in Tanzania all the more threatening since poultry farms in the country operate on a small to medium scale (URT, 2015). They are also typically managed by young people and women in peri-urban and rural areas. The farms are either located in the backyard grounds of many farmers' homes or they are semi-intensive in deep litter. Deep litter refers to a poultry farming system where poultry are maintained indoors on floors made of concrete covered with litters of sawdust or wood shavings (FAO, 2013). This farming system is mainly used in peri-urban areas. The impact of poultry diseases in these kinds of farms could lead to a total loss of poultry due to the lack of rapid diagnosis and treatment (URT, 2015).

The common poultry diseases that affect all farming systems include Salmonella, Infectious Coryza, Gumboro Pullorum, Newcastle, and Coccidiosis (Shirley et al., 2004; Desin et al., 2013; Mulisa et al., 2014; OIE, 2018). In addition, Salmonella disease is zoonotic. Globally, the costs associated with vaccination, mortality and control for coccidiosis poultry disease are estimated at £2 billion annually (Shirley et al., 2004). Early detection methods of the diseases have the potential to control them and improve poultry health. Salmonella, Coccidiosis and Newcastle diseases are diagnosed by laboratory procedures using fecal samples and it takes 3–4 days to get results. Access to these lab services by farmers is expensive and limited. Field extension officers and experienced farmers use clinical signs to diagnose the diseases in the field. The extension officers are the main source of information to farmers on poultry disease transmission, diagnosis, treatment and control (Msoffe et al., 2018). However, the extension officers are limited in number; one extension officer serves 10,000 to 20,000 farmers (Msoffe et al., 2018). There is a need to develop a cheap and practical diagnostics tool for poultry diseases for use by poultry farmers and extension officers. Such diagnostic tools can be developed using methods such as machine learning and deep learning.

Deep learning methods have been demonstrated to automate the disease diagnostics procedures for both human and livestock (Quinn et al., 2016; Zhuang et al., 2018; Okinda et al., 2019; Wang et al., 2019; Yadav and Jadhav, 2019). They have outperformed traditional imaging techniques in diagnostics of malaria, tuberculosis and intestinal parasite (Quinn et al., 2016). Deep learning has also been used in the diagnosis of crop diseases that attack cassava and bananas in East Africa (Owomugisha et al., 2014; Owomugisha and Mwebaze, 2016). With the help of deep learning, farmers have the potential to better diagnose poultry diseases and improve livestock health which would increase the production. Support Vector Machine (SVM) is one of the machine learning methods deployed to detect avian pox disease in poultry and diagnosis of hock burn prevalence in broiler chickens (Zhuang et al., 2018). The SVM approach has also been applied in monitoring egg production curves for commercial poultry farms and detection of broilers health status (Morales et al., 2016). The application of deep learning methods in disease diagnosis requires a dataset annotated by laboratory techniques such as Polymerase Chain Reaction (PCR).

The PCR is a molecular biology technique for rapid diagnostics. PCR method is used for detection and identification of pathogens through amplification of DNA sequences unique to the pathogen (Oliveira et al., 2003; Henderson et al., 2006). It saves time by reducing the testing process to several hours, compared to conventional methods of producing culture that takes over a week for diagnosis. However, the PCR tests are too expensive for poultry farmers to afford. In this study, we applied deep learning methods and PCR diagnostics to develop (i) a poultry diseases dataset and (ii) an end-to-end pipeline to diagnose poultry diseases of Coccidiosis, Salmonella, and Newcastle disease. Then, we proposed a suitable deep learning model for deployment of methods such as mobile application for early detection of poultry diseases at farm level.

## 2. Related work

### 2.1. Poultry diseases overview

Coccidiosis is caused by parasites of the genus *Eimeria* that affects the intestinal tracts of poultry. It is ranked as one of the leading sources of protozoan-caused deaths in poultry with *Eimeria tenella* (E.tenella) among the most pathogenic parasites (Lim et al., 2012). The typical diagnostic procedure involves counting the number of occysts (expressed as occysts per gram opg) in the feces and/or examining the intestinal tract to determine the lesion scores (Johnson and Reid, 1970; Grilli et al., 2018).

Salmonella spp are bacterial pathogens of the genus *Salmonella* that cause diseases in chickens, other domestic animals, and humans (Desin et al., 2013). *Salmonella pullorum* (SP) and *Salmonella gallinarum* (SG) pathogens cause pullorum disease and fowl typhoid in poultry respectively (Desin et al., 2013). *Salmonella enteritidis* (SE) and *Salmonella typhimurium* (ST) strains are associated with human infections transmitted through the food-chain of poultry and poultry products (Desin et al., 2013). Polymerase Chain Reaction (PCR) procedure is used for detection and identification of the various Salmonella strains (Oliveira et al., 2003).

Newcastle disease is an acute viral infection in poultry and other bird species caused by *avian paramyxovirus serotype 1* (APMV-1) viruses (Malik et al., 2021). Newcastle disease virus (NDV), APMV-1 is diagnosed by serology or virus isolation tests or real-time reverse-transcription PCR procedure (Wise et al., 2004). These diseases can be diagnosed early using machine learning and deep learning methods.

### 2.2. Machine learning methods in poultry

Recently, several studies have proposed machine learning methods as effective methods for detecting poultry diseases (Zhuang et al., 2018; Okinda et al., 2019; Wang et al., 2019). Wang et al. (2019) proposed an automated broiler digestive disease detector based on deep Convolutional Neural Network models, Faster R-CNN and YOLO-V3, to classify fine-grained abnormal broiler droppings images as normal and abnormal. Faster R-CNN achieved 99.1% recall and 93.3% mean average precision, while YOLO-V3 achieved 88.7% recall and 84.3% mean average precision on the testing data set. Wang et al. (2019)'s study contributes to the development of an automatic and non-contact model for identifying and classifying abnormal droppings in broilers suffering from digestive disease; however, an effective solution is required for early detection of poultry diseases.

In another study, Okinda et al. (2019) proposed a machine vision-based monitoring system for broiler chicken. Feature variables were extracted based on 2D posture shape descriptors and mobility features. Two sets of classifiers were then developed based on only the posture shape descriptors, and on all the feature variables. The Support Vector Machine (SVM) using a radial basis kernel function outperformed all the other models with an accuracy of 0.975. Despite the fact that the proposed system provides an early warning and prediction of an occurrence of disease continuously and non-intrusively, the system needs to be validated with different types of chicken breeds and infection types.

Support Vector Machine (SVM) were also used in another study that proposed an early warning algorithm for detecting sick broilers (Zhuang et al., 2018). The posture features of healthy and sick chickens were extracted, the eigenvectors were established, the postures of the broilers were analyzed by machine learning algorithms, and the diseased broilers were predicted. Accuracy rates of 84.2, 60.5, and 91.5% were obtained, but using all the features can yield an accuracy rate of 99.5%. Despite the fact that the study proposed a suitable method for small-sample learning and disease diagnosis, the focus was only based on a posture-based algorithm.

Therefore, fecal samples and images have potential in providing early mechanisms of diagnosing poultry diseases. Image data is unique in developing countries including Tanzania. Generally, in developing countries, there are low levels of literacy and multiple languages. The high penetration rates of mobile phones in developing countries means we can directly use the phones as a sensor (TCRA, 2021). There is a limitation of publicly available datasets with images of poultry fecal samples which hinders progress of research on early detection of poultry diseases. This study has developed a dataset that is available publicly for poultry diseases diagnostics.

## 3. Methods

### 3.1. The dataset

**Figure 1:**
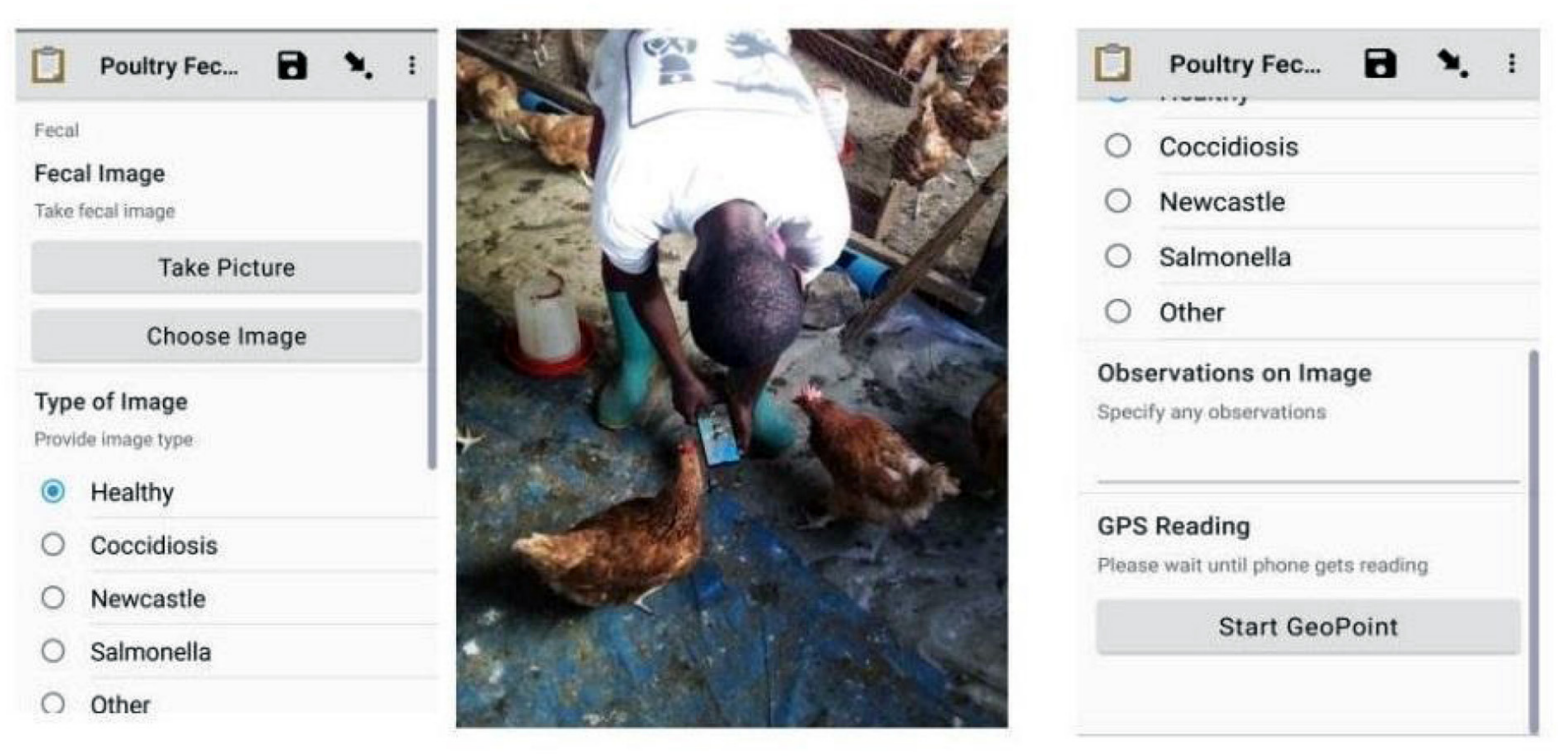
Veterinarians and researchers administering poultry data collection using ODK.

In this study, we developed a dataset of poultry fecal images for Coccidiosis, Newcastle, and Salmonella poultry diseases and healthy poultry. We gathered the fecal images from layers, cross, and indigenous breeds of chicken. These breeds are more vulnerable to diseases because they have a longer lifetime at the farms upto 18 months, compared to 5 weeks for broiler breeds. We collected the fecal images and fecal samples periodically between February 2020 and February 2021 from farms in Arusha and Kilimanjaro regions in Tanzania. We used the Open Data Kit (ODK) application installed on ordinary smartphones (Tecno, Infinix, Huawei, Samsung) to take photos of poultry fecal. ODK is a standard application for mobile data collection with support for geo-locations, images, audio clips, video clips, barcodes, numerical and textual answers (Hartung et al., 2010). Figure 1 shows the user interface of the ODK application and the enumerator collecting data in the field and Supplementary Figure 1 indicates the typical fecal images.

The data was stored on Google Drive. We organized the dataset in two groups. The first dataset consisted of 1255 poultry fecal images. In establishing this dataset, we collected fecal images and fecal samples from poultry farms. We stored the fecal samples in the freezer at −80 degrees Celsius in zip bags, labeled using barcodes. The barcodes captured using the ODK application link the samples and corresponding images. The PCR diagnostics procedure is expensive and thus we conducted it on only 16% of the total fecal samples at the molecular laboratory of Nelson Mandela African Institution of Science and Technology (NM-AIST). We used this to accurately detect and identify the diseases and establish labels on the images for the four (4) classes of Coccidiosis, Newcastle, Salmonella, and healthy poultry. The second dataset of the remaining 6,812 fecal images was established by only taking poultry fecal images and labeling them with the assistance from the animal health professionals i.e., veterinarian and field officer. The distribution of the dataset in the four classes is presented in Table 1.

**Table 1:** Dataset distribution of poultry fecal images for three diseased classes and one healthy class.

**Class - fecal images**	**Laboratory labeled -**	**Farm labeled -**
	**fecal images**	**fecal images**
Healthy	347	2,057
Salmonella	349	2,276
Coccidiosis	373	2,103
Newcastle	186	376
Total	1,255	6,812

Once all of the images were collected, we annotated the images for various computer vision tasks such as image segmentation, image object detection, and image classification. We began by first renaming the images in each class to be the class name and a number (i.e., “cocci.1.png” or “healthy.56.png”). We then stored the mapping of the original file name and new file name in a data frame along with other metadata about the image such as GPS coordinates. We stored the images in the original size in folders labeled by each class name. We annotated the images for the computer vision tasks of object detection and image segmentation using the LabelImg and LabelMe tools respectively (Russell et al., 2008; Tzutalin, 2015). In the labeling process of the farm-labeled fecal images, the color and shape of the images were the distinguishing feature between the different diseases. The fecal images for coccidiosis disease were predominantly dark brown with a flat shape; green color for Newcastle disease with a solid shape; white and loosely shaped for salmonella; gray, white, and solid shape were for the healthy label. It is likely to make labeling errors mainly for salmonella and healthy fecal images. The white color can be misinterpreted, the main difference is the texture: slimy and solid for salmonella and healthy respectively.

### 3.2. PCR diagnostics procedure

In order to label the fecal samples, we used existing primers from literature to amplify the target DNA/RNA on the poultry fecal samples for PCR. A primer is a short, single-stranded DNA used in PCR to define the region of the DNA that will be amplified. If the amplification of the target DNA or RNA is detected, it means the pathogen (virus or bacteria) was present in the sample (Menon et al., 1999). After identifying the primers, they were used for PCR diagnostics at the molecular laboratory of the Nelson Mandela African Institution of Science and Technology (NM-AIST). The fecal samples were stored at −80 degrees celsius and a sorting process was conducted to select the samples for PCR. The PCR diagnostics were conducted using reagents and kits from Zymo Research. The protocol is summarized as follows:

1. *DNA sample loading:*fecal samples were placed on Lysis tubes that contain bashing beads. Each sample was labeled with lab numbers.
2. *DNA extraction:* the samples were mechanically ruptured using Centrifuge 5430 R Eppendof machine and bead ruptor 24 repeatedly. A genomic buffer was added to each sample for binding the DNA. The filtered DNA was then suitable for PCR and other downstream applications.
3. *Amplification:* The samples were loaded on the PCR machine to amplify the targeted DNA using the identified primers. The machine was set to the corresponding annealing temperatures for primers of the corresponding disease.
4. *Quantification:* The DNA concentration was measured for every sample using a Spectrophotometer. The standard ratio for DNA concentration is in the range 1.8–2.0. Samples within this range of concentration were retained and considered passed on PCR. Samples out of this range were discarded.
5. *Detection:* We then confirmed the identified samples using the gel electrophoresis technique. We viewed the amplified DNA fragments on a visual dye on the gel.

### 3.3. Experimental design and setup

**Figure 2:**
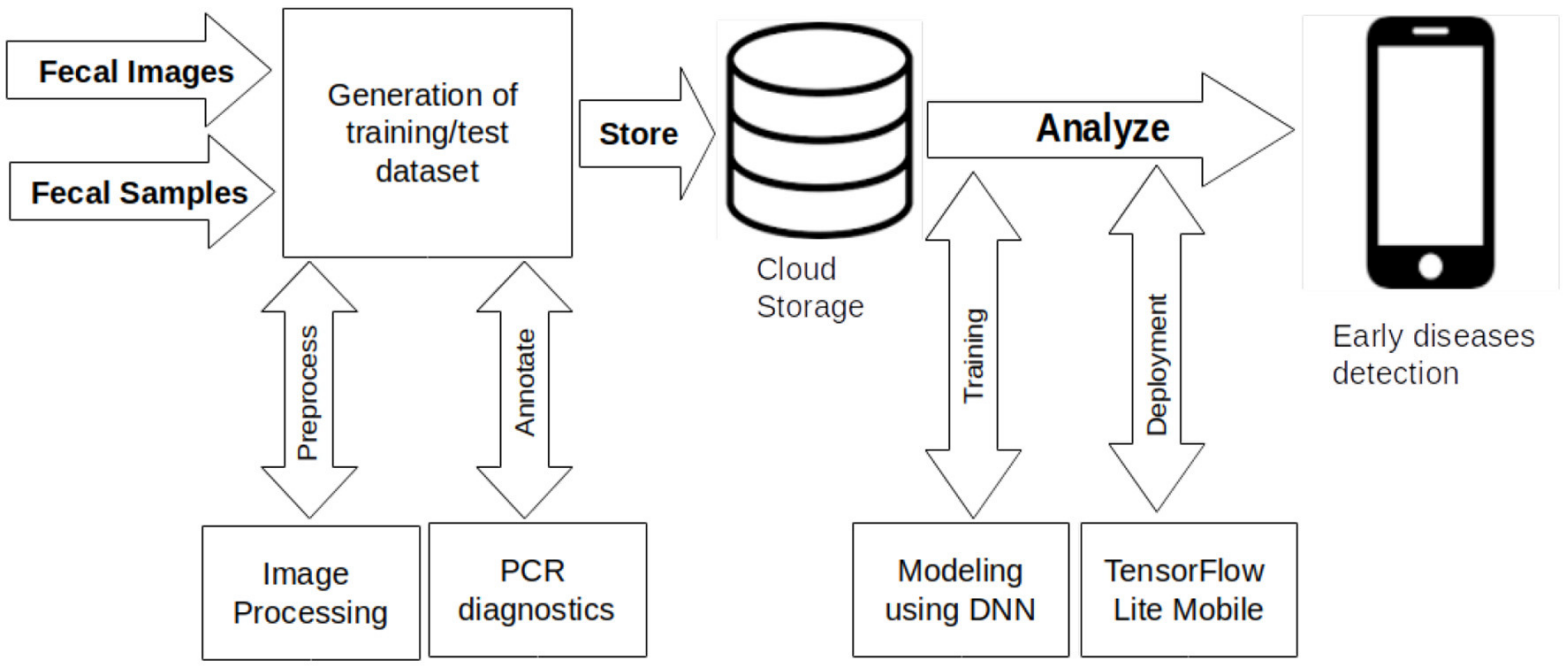
Automated poultry disease diagnostics research workflow.

The goal of our experimentation was to obtain a deep learning model for detecting three poultry diseases and to test its deployment on smartphones. The workflow indicated in Figure 2 guided the study through four steps: data generation, data annotation, modeling, and deployment. We generated a dataset of poultry fecal images for poultry diseases detection. The dataset was appropriate because the clinical signs of the poultry diseases are similar causing a challenge for farmers to differentiate the diseases. The fecal images display differences between the diseases that are clearer and easier to understand for farmers than the clinical signs. The clinical signs often are identified and validated by an agricultural support officer or a veterinarian. The clinical signs were not collected in this study. The color and shape of poultry fecal supplement clinical signs in detecting the diseases. The features of the data collected from farms included the color and shape of droppings and the number of chickens. The limitation of the dataset was missing clinical history data of the chickens. Farmers rarely keep these records. The annotation process of the dataset is detailed in the previous section. The next stage after annotation was modeling of images that were stored in the cloud. The modeling task was multi-class image classification for early detection of healthy, Newcastle, Coccidiosis and Salmonella poultry diseases.

In this study, we used Convolutional Neural Network (CNN) to train the images to classify three poultry diseases (Yadav and Jadhav, 2019). The CNN uses multiple feature extraction stages that learn representations automatically from the data (Goodfellow et al., 2016). We trained a CNN baseline model for multi-class image classification using Keras (Chollet, 2018; McDermott, 2020). Later, we used the transfer learning approach based on different CNN architectures since the images are limited in number for training from scratch for other CNN architectures (Chollet, 2018). We conducted the experiments using Google Colab Pro environment with a Tesla P100 GPU and 27.3 GB high-RAM runtime on TensorFlow library. We pre-processed the images using a combination of data augmentation techniques with good performance that included flipping and shifting (Howard, 2013; Pawara et al., 2017), with additional and varying augmentation techniques applied to specific model architectures (see Sections 3.5.1 to 3.5.4). In the following experiments, the dataset was split at 80 and 20% for training and testing respectively. Furthermore, 15% of the training data was used for validation. The dataset split used for baseline, pre-training, fine-tuning and testing is summarized in Supplementary Table 5 and hyper-parameters in Supplementary Table 4.

### 3.4. Baseline model

We pre-processed the image data using Keras *ImageDataGenerator* class (TensorFlow, 2022). It involved normalization and augmentation of the image data. We normalized the image pixel values in the range between 0 and 1 by the rescale argument of *ImageDataGenerator* class. The augmentation included: rotation at 90 degrees, varying the brightness in the range 0.1 to 0.7, shifting the width by 50%, and horizontally/vertically flipping images. The input images were resized to a target size of 128 × 128 pixels when loaded to memory using the *flow_from_directory* method of the *ImageDataGenerator* class.

After completing pre-processing of the images, we used a Keras model with the Sequential API to establish the building blocks of the CNN model. The input layer consisted of one convolutional block with 32 filters of window size 3 × 3 for input images of size 128 × 128. This was followed by four convolutional “blocks” each with (i) Convolutional layers, (ii) Max Pooling layer, and (iii) Dropout regularization of 0.2. All convolutional layers used the rectified linear unit (ReLU) activation function. The Fully-connected layer was constructed using Dense layers with a Softmax activation function since it is a multi-class problem. The model was trained at 100 epochs.

### 3.5. Experiments on transfer learning

The baseline experiment was a proof of concept that the real-world dataset of fecal images was feasible for classifying the three poultry diseases. However, the baseline model is not suitable for deployment on a mobile device. The baseline model learned from scratch had a limited number of features compared to transfer learning approaches, which provide many more features to improve the model performance and generalization (Hewitt and Gunes, 2018; Mehra et al., 2018). We then used the transfer learning approach for image classification to develop an end-to-end pipeline to diagnose the three poultry diseases. This involved four deep learning models with pre-trained weights: VGG16, InceptionV3, MobileNetV2, and Xception (Simonyan and Zisserman, 2014; Szegedy et al., 2016; Chollet, 2017; Sandler et al., 2018). We pre-trained and fine-tuned the deep learning models with a dataset of farm-labeled fecal images mixed with the laboratory-labeled fecal images. The goal of mixing the dataset for training was to increase the size and to obtain a more generalized performance. The test dataset consisted of only farm-labeled fecal images to reflect the types of datasets encountered in the field. The goal of fine-tuning was to improve the performance of the pre-trained models. We conducted another set of experiments with frozen batch normalization layers to further improve the accuracy of fine-tuned models and reduce overfitting (Ioffe and Szegedy, 2015).

The transfer learning workflow on pre-trained experiments using the four architectures mentioned above involved the following steps: (i) Instantiating a base model and loading pre-trained *Imagenet* weights into it; (ii) Freezing all layers in the base model by setting trainable = False. This will preserve the information they contain during training; (iii) Creating a new classification model (for 4 poultry classes) on top of the output of several layers from the frozen base model, by rebuilding the dense and softmax layers. This will turn the old features into predictions on new poultry fecal images dataset; (iv) Training the new model on the poultry fecal images dataset (Chollet, 2020). We used a similar workflow on fine-tuned experiments except step (ii) changed to setting trainable = True to unfreeze the layers at certain stages of the architecture, with the number of layers that were unfrozen varying across the different models. The aim of fine-tuning the models was re-training on the poultry fecal images dataset with a very low learning rate (reduces overfitting) to achieve improvements on model performance. Then on the last set of experiments to further improve the accuracy of fine-tuned models, we froze the batch normalization layers (contained in the base model) by setting trainable = False on these layers. This prevents the batch normalization layers from updating their batch statistics (mean and variance) during training and will not destroy the representations learned by the model up to that step (Chollet, 2020).

#### 3.5.1. VGG16

We used the VGG16 model, which is a convolutional neural network trained on a subset of the ImageNet dataset (Simonyan and Zisserman, 2014). We first pre-processed the fecal images dataset by performing normalization and augmentation using the *ImageDataGenerator*. All the numerical values in our input images were normalized to a value in the range [−1, 1]. In addition to flipping the images vertically and horizontally, we augmented them by shifting the width and height by 50%, rotation range of 90 degrees and varied the brightness in the range 0.1 to 0.7 (McDermott, 2021). Augmentation increases variance across images (Shorten and Khoshgoftaar, 2019). We specified the validation split of the training data to be used for validation at the end of each epoch. We provided the target_size of 224 × 224 pixels, the required size for the VGG16 model input and a batch size of 64. We performed transfer learning without fine-tuning by freezing all the pre-trained layers of the base model. We created the new Fully-Connected layer using the fecal images inputs by flattening the outputs of the base model, followed by a Dense layer with 4,096 neurons then another Dense layer of 1,072 neurons. Both dense layers used ReLU activation function. Then we applied a dropout of 0.2 and a Dense layer to obtain the final prediction of 4 classes and Softmax activation. We then fine-tuned the VGG16 model by unfreezing the last two pre-trained layers and lowering the learning rate to help the Fully-Connected layer learn robust patterns previously learned. In the third experiment, we froze the BatchNormalization layers of the fine-tuned model to improve the accuracy (Ioffe and Szegedy, 2015; Chollet, 2020).

#### 3.5.2. InceptionV3

We conducted another experiment using the InceptionV3 model (Szegedy et al., 2016). The pre-processing of the fecal images involved normalization of all the pixel values in input images to a value in the range [0, 1]. The augmentations techniques applied to the training fecal images included shifting the height and width by 20%; rotation range of 40 degrees; shear and zoom range of 20%. We loaded the data for training with a batch size of 64 and a target size of 299 × 299 pixels. In the base pre-training, all convolutional InceptionV3 layers were frozen (non-trainable). Then, we added a global spatial average pooling layer followed by a Dense layer (1,024 neurons) with ReLU activation function. The output Dense layer had a size of four, equivalent to the number of classes of fecal images on Softmax activation function. We then trained only the top layers. In the fine-tuning experiment, we froze the entire network except the mixed8 layer for feature extraction in InceptionV3. The mixed8 is a 8 × 8 × 1280 convolution, the top layer of InceptionV3 model. We re-compiled the model and trained at a lower learning rate using the hyper-parameters indicated on Supplementary Table 4. In the third experiment, we froze the BatchNormalization layer of the fine-tuned model.

#### 3.5.3. MobileNetV2

The MobileNetV2 architecture is one of the TensorFlow pre-trained models for image classification (Sandler et al., 2018). We used the Keras ImageDataGenerator class to load the images from sub-directories with a batch size of 64 and target shape of 224 × 224 in all three experiments. The image data augmentation was vertical flip, brightness range of [0:2; 1], and random rotation of 45 degrees. We normalized the pixel values in the range [0; 1] by setting the rescale = 1.0/255. attribute in the ImageDataGenerator class. We shuffled the training data (Shuffle=True) and the validation and test data was not shuffled (Shuffle=False). In the first experiment, we pre-trained the base model by freezing all the layers and added a new classifier. The classifier consisted of a global average pooling layer, followed by a dropout of 0.2 for regularization and the Dense layer for prediction of size 4 and Softmax activation function. In the fine-tuning experiment, we unfroze the base model and retrained with the same classifier. In further improving the accuracy, we froze the BatchNormalization layers of the fine-tuned model in the third experiment. The classifier remained the same as in the previous experiments above.

#### 3.5.4. Xception

In this experiment, we used a different pre-trained model on the TensorFlow framework, the Xception model (Chollet, 2017). The image pre-processing step using the ImageDataGenerator class involved normalization in the range [0, 1], brightness in the range [0.1, 0.7] and image data augmentation of horizontal and vertical flip. We provided the target size of 299 × 299 pixels, for the Xception model input and a batch size of 64. The training and validation data were shuffled and the test set was not shuffled. We used a similar approach to the MobileNetV2 model in compiling the model with the hyper-parameters indicated in Supplementary Table 4 in the pre-training and two fine-tuning experiments. In the fine-tuning experiments, we unfroze 132 layers of the base model for training.

## 4. Results and discussion

We developed a poultry fecal images dataset to classify three poultry diseases. The fecal images dataset in Table 1 has four classes of Coccidiosis, Salmonella, Newcastle disease and healthy. We used the dataset to train the Convolutional Neural Network (CNN) architectures presented in the previous sections. The model performance for transfer learning experiments and the baseline model are indicated in Table 2.

**Table 2:** Model performance results—accuracy and F1 scores (from frozen batchnorm).

**Models**	**Accuracy (without fine-tuning)** **%**	**Accuracy (fine-tuning)** ** %**	**Accuracy (fine-tuning), frozen batchnorm** **%**	**F1 score, frozen batchnorm** ***{cocci, healthy, ncd, salmo}***
Baseline CNN	83.06			{0.92, 0.86, 0.14, 0.81}
VGG16	85.85	94.65	95.01	{0.98, 0.95, 0.77, 0.95}
InceptionV3	94.79	94.94	95.45	{0.98, 0.94, 0.77, 0.95}
MobileNetV2	87.46	97.14	98.02	{0.99, 0.97, 0.94, 0.99}
Xception	88.27	97.36	98.24	{0.97, 0.98, 0.98, 0.99}

We used accuracy as one of the performance metrics on the experiments of the five models: baseline CNN, VGG16, InceptionV3, MobileNetV2 and Xception. In training the models without fine-tuning, the baseline model had the lowest accuracy of 83.06% on test data. The InceptionV3 had the highest accuracy of 94.79%. In fine-tuning the transfer learning models, the accuracy improved on test data for the four models. The accuracy on fine-tuned models for VGG16 model was 94.65%, InceptionV3 at 94.94%, MobileNetV2 at 97.14% and Xception model 97.36%. The accuracy of fine-tuned models with frozen BatchNormalization layers improved the accuracy of fine-tuned models. The accuracy were: VGG16 model was 95.01%, InceptionV3 at 95.45%, MobileNetV2 at 98.02% and Xception model 98.24%. When referring to the loss curves in Supplementary Figures 4, 5, the accuracy for MobileNetV2 and Xception on the Batchnorm frozen experiment both seem to be around 0.75–0.85 after 10 epochs, and loss seems to be around 0.65–0.75. MobileNetV2 seems to stabilize validation loss at 0.6 around 20–30 epochs with no sign of over-fitting. The validation loss of Xception model (Supplementary Figure 5) is unstable above 15 epochs suggesting some overfitting. In addition, model size of MobileNetV2 batchnorm frozen model is almost 10 times smaller (26 MB) than the Xception model (238 MB). The trainable parameters for MobileNetV2 were 2.19 m and Xception had 20.76 m parameters. All Convolution and Separable Convolution layers in Xception are followed by batch normalization (Chollet, 2017). The model is batch normalization heavy (Ioffe and Szegedy, 2015). In the fine-tuning experiments, we unfroze the layers of the base model by setting trainable=True and disabled weight updates by setting training=False. The batch normalization weights were not updated in the learning phase and this highly increased the accuracy compared to the without fine-tuning results. When we froze the batch normalization layers, the layers were in inference mode (during training). When the batchnorm layers are in inference mode in training (base_model.trainable=True and base_model(inputs, training=False)), their internal state will not change during training, the trainable weights will not be updated. The accuracy slightly increased by 0.88% for both MobileNetV2 and Xception models in frozen batchnorm experiments.

We determined other performance metrics of the classifiers; the baseline CNN and the fine-tuned transfer learning models with frozen batch normalization layers. These classifiers have higher accuracy than the ones trained without fine-tuning. Supplementary Table 6 reports the precision, recall, and F1 score of the classifiers from the final experiment with frozen Batchnorm. Our classifiers achieved F1 score greater than 75% for all four classes in four models. The highest F1 score indicates the highest number of correct predictions. The F1 score of the cocci class was highest in the classifiers: 91.61% (baseline CNN), 97.85% (VGG16), 97.79% (InceptionV3), 99.17% (MobileNetV2), and 98.71% (Xception). The precision of Xception is the highest for two classes (Salmonella and healthy), MobileNetV2 for the Coccidiosis class and InceptionV3 was highest for the Newcastle disease class. The Xception model has the ability to correctly predict Salmonella at 99.33% and healthy at 97.84%. MobileNetV2 model can correctly predict Coccidiosis disease at 98.82%. The InceptionV3 model can predict Newcastle disease at 100% from the poultry fecal images. The model recall is 100% on InceptionV3 and Xception models for the cocci class meaning all relevant Coccidiosis images are correctly classified by the model. The recall on the MobileNetV2 and Xception models were above 85% for all four classes. The lowest recall score on Inception V3 model for Newcastle disease was 62.7%.

We also explored the visualizations of the feature maps extracted from first convolutional layer close to the input image and on layers close to the output. These are indicated in Supplementary Figure 6. It was observed that the feature maps extracted close to the input detect fine-grained detail of the fecal images, whereas feature maps close to the output have more general features of the images.

Given the lighter weight of the trained MobileNetV2 and its superior ability to generalize, as evident by the lack of over-fitting in its validation loss (Supplementary Figure 4), we recommend deploying this model for early detection of poultry diseases at the farm level. The workflow will involve converting the MobileNetV2 model to TensorFlow Lite format before running it on smartphones (Android or iOS devices) (Moroney, 2020). The conversion to TensorFlow Lite format has minimum impact on the model accuracy and reduces the model size (Moroney, 2020). The accuracy of the test set for MobileNetV2 and Xception models on the BatchNorm frozen experiment indicates a very small difference, with Xception having higher accuracy by 0.22%. The other metrics of precision, recall, and F1 score on MobileNetV2 are all above 87%. Supplementary Figure 7 indicates the inference of MobileNetV2 and Xception models. The inference for the three poultry diseases, Coccidiosis, Salmonella, Newcastle disease, and healthy poultry will be conducted on the field using a mobile device. The application will run offline which guarantees usage without the internet.

## 5. Conclusion

This paper presented a deep learning model for detecting poultry diseases. A deep Convolutional Neural Network (CNN) model was developed to diagnose healthy and unhealthy poultry fecal images. We trained on different deep convolutional neural network architectures that included a baseline CNN, VGG16, InceptionV3, MobileNetV2, and Xception. We trained the models with farm labeled and laboratory labeled fecal images and later tested on farm labeled fecal images. After comparison with other models, the MobileNetV2 model showed the highest potential for deployment on smartphones, to be used as a diagnostic tool that farmers can use to distinguish diseased from healthy poultry based on fecal images. The field testing of the deployed model will be conducted in collaboration with a network of farmers' groups in Tanzania. This proposed solution will be less expensive for farmers than PCR diagnostics tests in the laboratory. Even though this model was trained using data from several farms in northern Tanzania, we expect it will be able to perform effectively elsewhere in Tanzania, where the poultry disease distribution is the same. We do not expect the model to be impacted by environment and distribution shift.

In providing technical approaches associated with poultry health, this study contributed a new dataset on poultry fecal samples which can be used by other researchers in the field (Machuve et al., 2021a,b). The study was limited to the classification approach which has been used to classify healthy and unhealthy images. As part of future work, object detection for real time monitoring and segmentation may be applied in order to carry out real time prediction and quantification. Further, we recommend training and field testing of the solution with farmers.

## Data availability statement

The datasets generated for this study can be found in the ZENODO REPOSITORY. The dataset consists of poultry fecal images that are:

- Farm labeled fecal images https://zenodo.org/record/4628934#.YtDNzOxBy1u
- Laboratory labeled fecal images https://zenodo.org/record/5801834#.YtDN9-xBy1t.

## Ethics statement

The animal study was reviewed and approved by Kibong'oto Infectious Diseases Hospital–Nelson Mandela African Institution of Science and Technology–Centre for Educational Development in Health, Arusha (KNCHREC). The project was conducted under the Research Ethical Clearance Certificate no: KNCHREC00027 issued on 14th February, 2020 for one year. Written informed consent was obtained from the owners for the participation of their animals in this study. Written informed consent was obtained from the individual(s) for the publication of any potentially identifiable images or data included in this article.

## Author contributions

DM oversaw data collection and labeling of the poultry dataset in Tanzania. DM and EN annotated the images, trained the deep learning models, and took the lead in writing the manuscript. All authors provided critical feedback and helped shape the research, analysis and manuscript, and contributed to the article and approved the submitted version.

## Funding

This work was carried out with the aid of a grant from UNESCO and the International Development Research Center, Ottawa, Canada. The views expressed herein do not necessarily represent those of UNESCO, IDRC, or its Board of Governors.

## Conflict of interest

The authors declare that the research was conducted in the absence of any commercial or financial relationships that could be construed as a potential conflict of interest.

## Publisher's note

All claims expressed in this article are solely those of the authors and do not necessarily represent those of their affiliated organizations, or those of the publisher, the editors and the reviewers. Any product that may be evaluated in this article, or claim that may be made by its manufacturer, is not guaranteed or endorsed by the publisher.
