# Corrigendum: Poultry diseases diagnostics models using deep learning

**DOI:** 10.3389/frai.2022.1016695

**Published:** 2022-09-01

**Authors:** Dina Machuve, Ezinne Nwankwo, Neema Mduma, Jimmy Mbelwa

**Affiliations:** ^1^Department of IT Systems Development and Management, Nelson Mandela African Institution of Science and Technology, Arusha, Tanzania; ^2^Department of Electrical Engineering and Computer Science, University of California, Berkeley, Berkeley, CA, United States; ^3^Department of Computer Science and Engineering, University of Dar es Salaam, Dar es Salaam, Tanzania

**Keywords:** deep learning, agriculture, poultry disease diagnostics, dataset, image classification

In the published article, there was an error in the **Funding** statement. The funding statement did not use the text that is on the project agreement with the funder. The funding statement was displayed as: “The research project was funded by two grants: (1) The 2019 Early Career Fellowship Program of the Organization for Women in Science for the Developing World (OWSD) and (2) The International Development Research Center (IDRC) with IDRC Grant Number: 109187-002 through the 2020 IndabaX-AI4D Innovation Grant.”

The correct **Funding** statement appears below.

## Funding

This work was carried out with the aid of a grant from UNESCO and the International Development Research Center, Ottawa, Canada. The views expressed herein do not necessarily represent those of UNESCO, IDRC, or its Board of Governors.

The authors apologize for this error and state that this does not change the scientific conclusions of the article in any way. The original article has been updated.

## Publisher's note

All claims expressed in this article are solely those of the authors and do not necessarily represent those of their affiliated organizations, or those of the publisher, the editors and the reviewers. Any product that may be evaluated in this article, or claim that may be made by its manufacturer, is not guaranteed or endorsed by the publisher.

