# Supplementary material for: Poultry diseases diagnostics models using deep learning
Source: Front Artif Intell. 2022 Aug 1;5:733345. doi: 10.3389/frai.2022.733345 (PMC9376463; doi:10.3389/frai.2022.733345)
Supplement: Supplementary file 1 [file Data_Sheet_1.PDF]

Supplementary Material

1 DATASHEETS FOR DATASET: POULTRY FECAL IMAGES

1.1 Motivation for Dataset Creation

The poultry fecal images dataset was created to contribute in the study of poultry diseases diagnostics. The fecal images target the diagnostics of Coccidiosis, Salmonella and Newcastle poultry diseases. We are motivated in developing end to end tools to help farmers diagnose the diseases and improve poultry health. The dataset was created to facilitate image classification task.

1.1.1 What (other) tasks could the dataset be used for? Are there obvious tasks for which it should not be used?

The poultry fecal images dataset can be used for various computer vision tasks such as image segmentation, image object detection, and image classification. These tasks promise to reduce bias from the background on the images when training on deep convolutional neural network.

1.1.2 Has the dataset been used for any tasks already? If so, where are the results so others can compare (e.g., links to published papers)?

- A Poster presentation at the Mechanism Design for Social Good (MD4SG'19 and MD4SG'20) Workshop:
  1. ”Towards Automated Poultry Diseases Diagnostics” by Ezinne Nwankwo and Dina Machuve.
  2. “Image-based Poultry Disease Detection Using DCNN” by Hope Mbelwa and Dina Machuve.
- Two Published datasets with usage reflected by the number of downloads:
  1. Machuve, Dina; Nwankwo, Ezinne; Mduma, Neema; Mbelwa, Hope; Maguo, Evarist; & Munisi, Charles. (2021). Machine Learning Dataset for Poultry Diseases Diagnostics (Version 2) [Data set]. Zenodo. `http://doi.org/10.5281/zenodo.4628934`
  2. Machuve, D., Nwankwo, E., Lyimo, E., Maguo, E., and Munisi, C. (2021a). Machine Learning Dataset for Poultry Diseases Diagnostics - PCR Annotated (Version 3). [Data set]. Zenodo. `https://doi.org/10.5281/zenodo.5801834`

1.1.3 Who funded the creation of the dataset? If there is an associated grant, provide the grant number.

The research project was funded by:

- The Organization for Women in Science for the Developing World (OWSD) Early Career Fellowship Program (2019-2021)
- The International Development Research Centre (IDRC) with IDRC Grant Number: 109187-002 through the 2020 IndabaX-AI4D Innovation Grant.

1.2 Dataset Composition

1.2.1 What are the instances? (that is, examples; e.g., documents, images, people, countries) Are there multiple types of instances? (e.g., movies, users, ratings; people, interactions between them; nodes, edges)

Each instance is a set of images labeled based on the classes which are Healthy, Salmonella, Newcastle and Coccidiosis.

1.2.2 Are relationships between instances made explicit in the data (e.g., social network links, user/movie ratings, etc.)?

There are no known relationships between instances except for the fact that they are all poultry fecal samples.

1.2.3 How many instances of each type are there?

The dataset consists of 1255 laboratory labeled fecal images (Healthy = 347, Salmonella = 349, Coccidiosis = 373, Newcastle = 186) and 6812 farm labeled fecal images (Healthy = 2057, Salmonella = 2276, Coccidiosis = 2103, Newcastle = 376)

1.3 Data Collection Process

1.3.1 How was the data collected? (e.g., hardware apparatus/sensor, manual human curation, software program, software interface/API; how were these constructs/measures/methods validated?)

The poultry fecal images for this dataset were collected using camera on mobile phones from healthy and chickens with three diseases. Open Data Kit (ODK) application installed on ordinary smartphones (Tecno, Infinix, Huawei, Samsung) that were used to take photos of poultry fecal.

In creating the laboratory labeled dataset, we collected both poultry fecal samples and fecal images. The image of the fecal sample was first taken using the ODK application then the fecal sample was collected. The samples were stored on a plastic sachet with a barcode to identify it with the corresponding image. The fecal samples were stored in the laboratory freezer at -80 degrees celsius. Polymerase Chain Reaction (PCR) diagnostics procedure was conducted to accurately label the fecal samples and the corresponding images.

1.3.2 Who was involved in the data collection process? (e.g., students, crowdworkers) How were they compensated? (e.g., how much were crowdworkers paid?)

The data collection process involved the project team members including Masters' student and the veterinarian who was in charge of the poultry and overall field activities. The PCR diagnostics procedure involved a Life Sciences laboratory technician at the Molecular Biology laboratory at the Nelson Mandela African Institution of Science and Technology in Arusha, Tanzania.

1.3.3 Over what time-frame was the data collected? Does the collection time-frame match the creation time-frame?

The data was collected in the range of twelve months (1 year), periodically between February 2020 and February 2021 from farms in Arusha and Kilimanjaro regions in northern Tanzania.

1.3.4 How was the data associated with each instance acquired? Was the data directly observable (e.g., raw text, movie ratings), reported by subjects (e.g., survey responses), or indirectly inferred/derived from other data (e.g., part of speech tags; model-based guesses for age or language)? If the latter two, were they validated/verified and if so how?

The names for each disease in the dataset were determined by looking at the caption associated with the fecal sample. There was a possibility of assigning incorrect diseases especially when the original caption was incorrect.

1.3.5 Does the dataset contain all possible instances? Or is it, for instance, a sample (not necessarily random) from a larger set of instances?

The dataset contains all possible instances.

1.3.6 If the dataset is a sample, then what is the population? What was the sampling strategy (e.g., deterministic, probabilistic with specific sampling probabilities)? Is the sample representative of the larger set (e.g., geographic coverage)? If not, why not (e.g., to cover a more diverse range of instances)? How does this affect possible uses?

The poultry fecal images dataset is a sample of labeled images collected from the field. The intention was to have the dataset of poultry diseases diagnostics with consideration of three identified diseases which are mainly affecting productivity.

1.3.7 Is there information missing from the dataset and why? (this does not include intentionally dropped instances; it might include, e.g., redacted text, withheld documents) Is this data missing because it was unavailable?

The dataset contains all required information.

1.4 Data Preprocessing

1.4.1 What preprocessing/cleaning was done? (e.g., discretization or bucketing, tokenization, part-of-speech tagging, SIFT feature extraction, removal of instances, processing of missing values, etc.)

The following steps were taken to process the data:

- Gathering raw images: First the poultry fecal images were collected using Open Data Kit (ODK) application installed on ordinary smartphones.
- Eliminating duplicate images: The identified duplicate images were removed but a very small number (that were not initially identified) might still exist in the dataset. The number of remaining duplicates should be small enough so as not to significantly impact training/testing. The dataset contains distinct images that are not defined to be duplicates but are extremely similar.
- Labeling : The images were labelled to indicate the belonging class (healthy, Salmonella, Newcastle, Coccidiosis).
- Curation: The images were annotated for various computer vision tasks such as image segmentation, image object detection, and image classification.
- Renaming: The images in each class were renamed to comprise class name and a number (i.e. “cocci.1.png” or “healthy.56.png”).

1.4.2 Was the “raw” data saved in addition to the preprocessed/ cleaned data? (e.g., to support unanticipated future uses)

The raw unprocessed data (consisting of labeled images of poultry fecal samples) is saved.

1.4.3 Is the preprocessing software available?

Software used to process data is open source and publicly available on `https://github.com/ezinne359/AI4D-Poultry-Dataset`

1.4.4 Does this dataset collection/processing procedure achieve the motivation for creating the dataset stated in the first section of this datasheet?

There some potential limitations in the dataset which might led to bias:

- The dataset does not contain many images that occur under extreme (or very low) lighting conditions.
- The dataset does not contain many images that were collected by using high quality smartphone cameras.

1.5 Dataset Distribution

1.5.1 How is the dataset distributed? (e.g., website, API, etc.; does the data have a DOI; is it archived redundantly?)

The dataset is publicly available on the Zenodo platform with the following DOI:

- https://doi.org/10.5281/zenodo.4628934
- https://doi.org/10.5281/zenodo.5801834

1.5.2 When will the dataset be released/first distributed? (Is there a canonical paper/reference for this dataset?)

The dataset was released on February 3, 2021.

1.5.3 What license (if any) is it distributed under? Are there any copyrights on the data?

This dataset licensed under a Creative Commons Attribution 4.0 International

1.5.4 Are there any fees or access/export restrictions?

There are no fees or restrictions.

1.6 Dataset Maintenance

1.6.1 Who is supporting/hosting/maintaining the dataset? How does one contact the owner/curator/manager of the dataset (e.g. email address, or other contact info)?

The dataset is hosted on Zenodo and at the Nelson Mandela African Institution of Science and Technology (NM-AIST) and all comments can be sent to: dina.machuve@nm-aist.ac.tz

1.6.2 Will the dataset be updated? How often and by whom? How will updates/revisions be documented and communicated (e.g., mailing list, GitHub)? Is there an erratum?

All changes to the dataset will be announced through the mailing list on the host server.

1.6.3 If others want to extend/augment/build on this dataset, is there a mechanism for them to do so? If so, is there a process for tracking/assessing the quality of those contributions. What is the process for communicating/distributing these contributions to users?

Other researchers are allowed to extend this dataset, interested researchers should send an email to dina.machuve@nm-aist.ac.tz for further details.

1.7 Legal & Ethical Considerations

The project was conducted under the Research Ethical Clearance Certificate no: KNCHREC00027 issued on 14th February, 2020 with one year validity until 13th February, 2021. It was issued by the Kibong'oto Infectious Diseases Hospital – Nelson Mandela African Institution of Science and Technology – Center for Educational Development in Health, Arusha (KNCHREC).

1.7.1 Does the dataset contain information that might be considered sensitive or confidential? (e.g., personally identifying information)

The dataset does not contain any sensitive or confidential information.

2 SUPPLEMENTARY TABLES AND FIGURES

2.1 Tables

Name	Primer	Sequences (5'–3')	Amplicon size (bp)	An(°C)	Reference
M	4100F/<br>4220R	5'-AGTGATGTGCTCGGACCTTC-3'<br>5'-CCTGAGGAGAGGCATTTGCTA-3'	121	58	Wise et al., 2004

Table S1. Set of primers used for amplification of partial M gene of Newcastle disease virus

Name	Primer	Sequences (5'–3')	Amplicon size (bp)	An(°C)	Reference
Eimeria spp.18S rDNA	ERIB1/<br>ERIB10	ACCTGGTTGATCCTGCCAG<br>CTTCCGCAGGTTCACCTACGG	1790	57	Kumar et al., 2014

Table S2. Set of primers used for amplification of Eimeria genus for Coccidiosis

Name	Primer	Sequences (5'–3')	Amplicon size (bp)	An(°C)	Referer
invA	139/<br>141	5'-GTGAAATTATCGCCACGTTCG GGCAA-3'<br>5'-TCATCGCACCGTCAA AGG AACC-3'			Zahraei, e 2006

Table S3. Set of primers used for amplification of partial invA gene of Salmonella

Parameter	Model				
	**Baseline**	**VGG16**	**InceptionV3**	**MobileNetV2**	**Xception**
Epochs	100	100	100	100	100
Batch size	32	64	64	64	64
Optimizer	RMSprop	Adam	RMSprop	Adam	Adam
Learning rate	1e-3	1e-3 (pre-training)<br>1e-4 (fine-tuning)	1e-3 (pre-training)<br>1e-3 (fine-tuning)	1e-3 (pre-training)<br>1e-4 (fine-tuning)	1e-3 (pre-training)<br>1e-4 (fine-tuning)
Early stopping	10 epochs	10 epochs	10 epochs	10 epochs	10 epochs

Table S4. The hyper-parameters used for training

	Baseline	VGG16	InceptionV3	MobileNetV2	Xception
Training	5464	5464	5464	5464	5464
Validation	963	963	963	963	963
Testing	1364	1364	1364	1364	1364

Table S5. The number of fecal images after pre-processing in five deep learning models

2.2 Figures

Class	Baseline CNN		
	**Precision**	**Recall**	**F1-Score**
cocci	0.855670	0.985748	0.916115
healthy	0.783838	0.944039	0.856512
ncd	0.176471	0.120000	0.142857
salmo	0.963964	0.702407	0.812658

Class	VGG16		
	**Precision**	**Recall**	**F1-Score**
cocci	0.985542	0.971496	0.978469
healthy	0.940476	0.961071	0.950662
ncd	0.770270	0.760000	0.765101
salmo	0.956044	0.951860	0.953947

Class	InceptionV3		
	**Precision**	**Recall**	**F1-Score**
cocci	0.956818	1.000000	0.977933
healthy	0.898901	0.995134	0.944573
ncd	1.000000	0.626667	0.770492
salmo	0.990521	0.914661	0.951081

Class	MobileNetV2		
	**Precision**	**Recall**	**F1-Score**
cocci	0.988208	0.995249	0.991716
healthy	0.971223	0.985401	0.978261
ncd	0.942857	0.880000	0.910345
salmo	0.986755	0.978118	0.982418

Class	Xception		
	**Precision**	**Recall**	**F1-Score**
cocci	0.974537	1.000000	0.987104
healthy	0.978417	0.992701	0.985507
ncd	0.984848	0.866667	0.921986
salmo	0.993318	0.975930	0.984547

Table S6. Model Metrics: Precision, Recall, F1 Scores for different Model Architectures

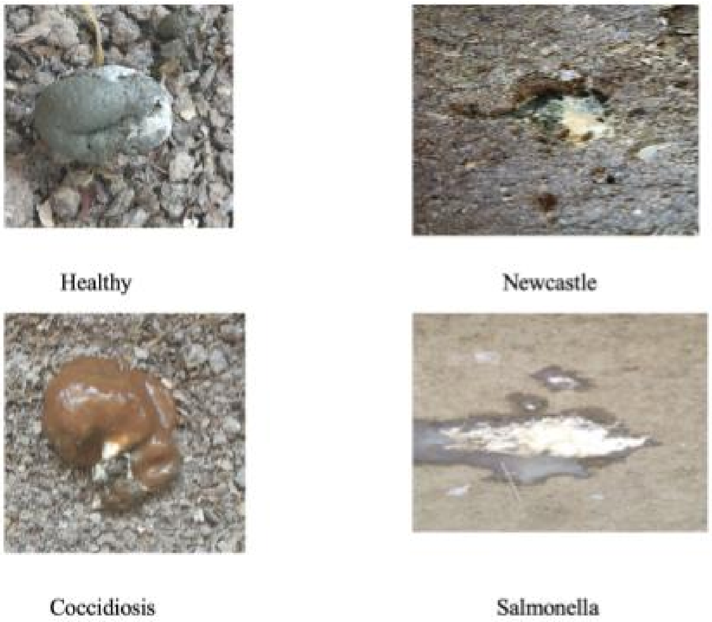

Figure S1. Poultry fecal images for a) Healthy b) Newcastle c) Coccidiosis d) Salmonella Classes

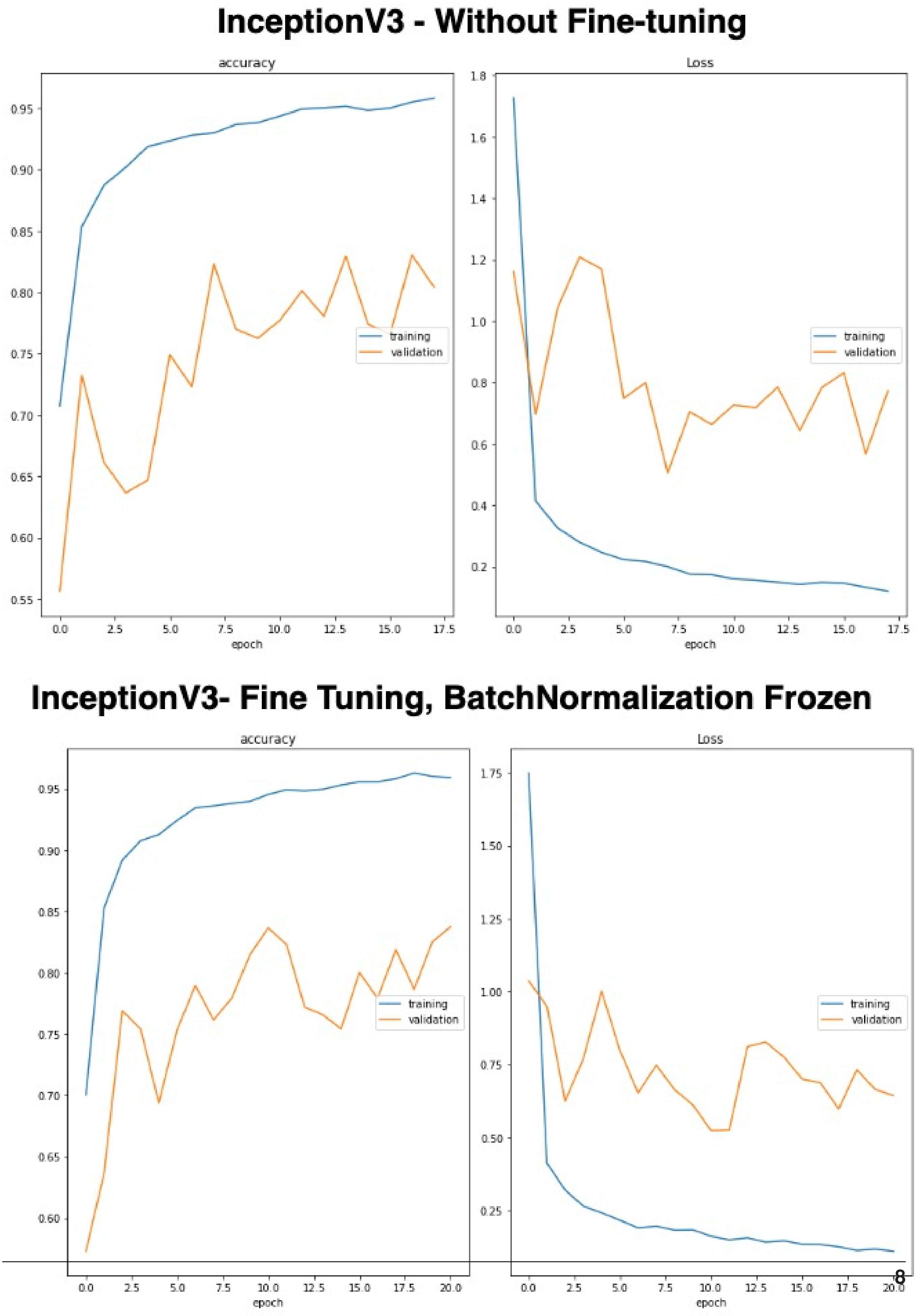

Figure S2. Training and validation curves (accuracy and loss) for the InceptionV3 classifier

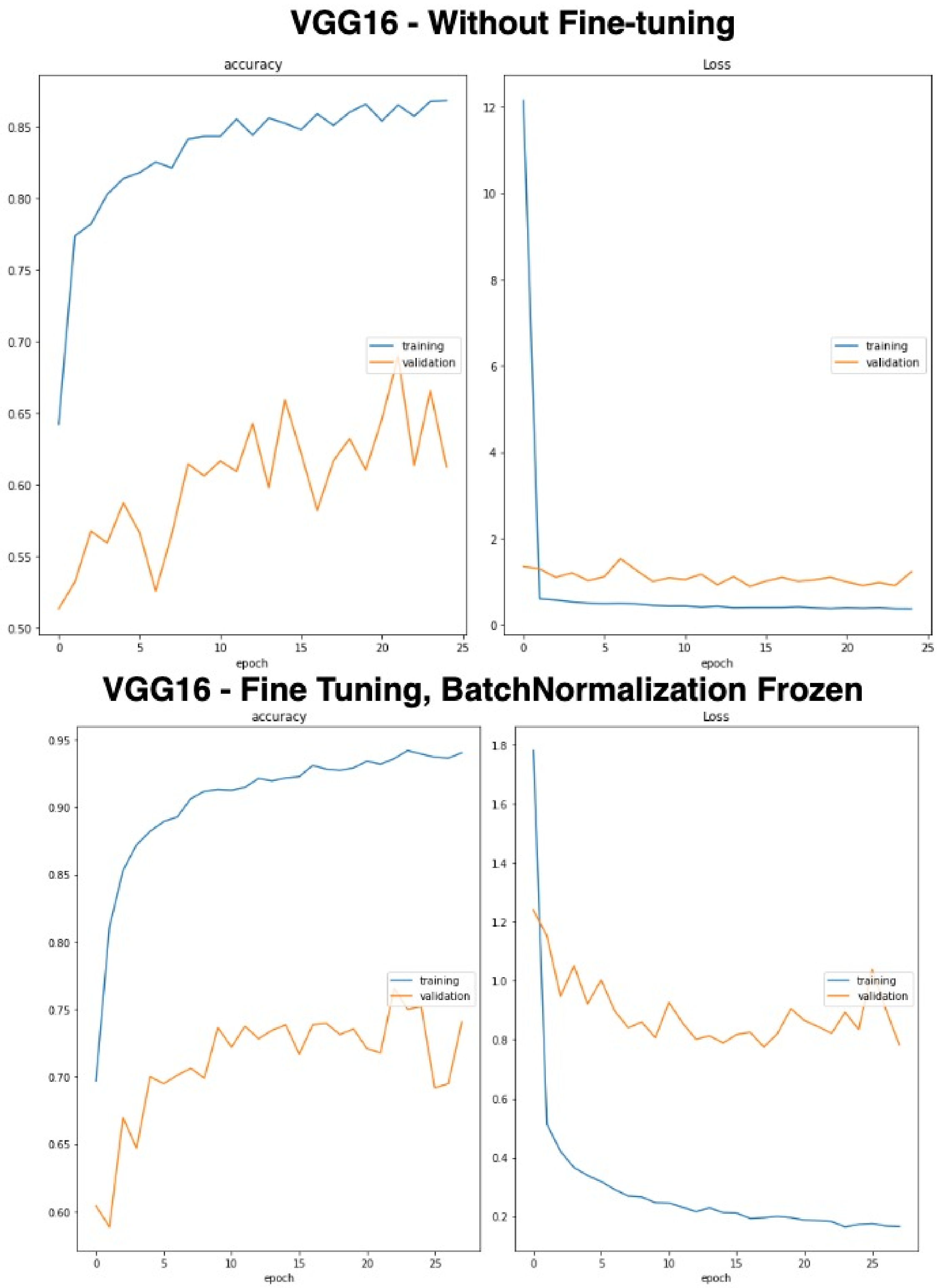

Figure S3. Training and validation curves (accuracy and loss) for VGG16 classifier

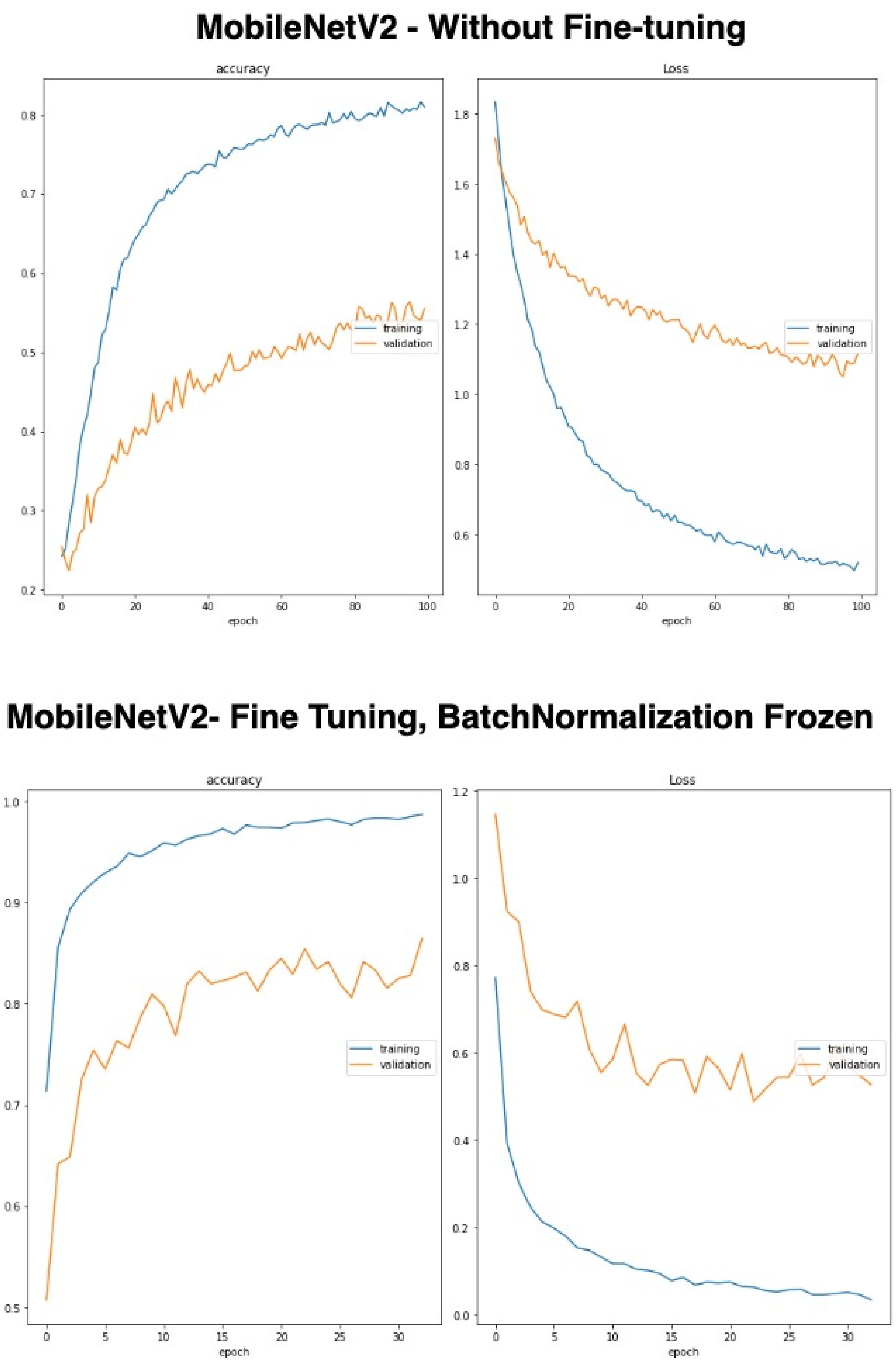

Figure S4. Training and validation curves (accuracy and loss) for MobileNetV2 classifier

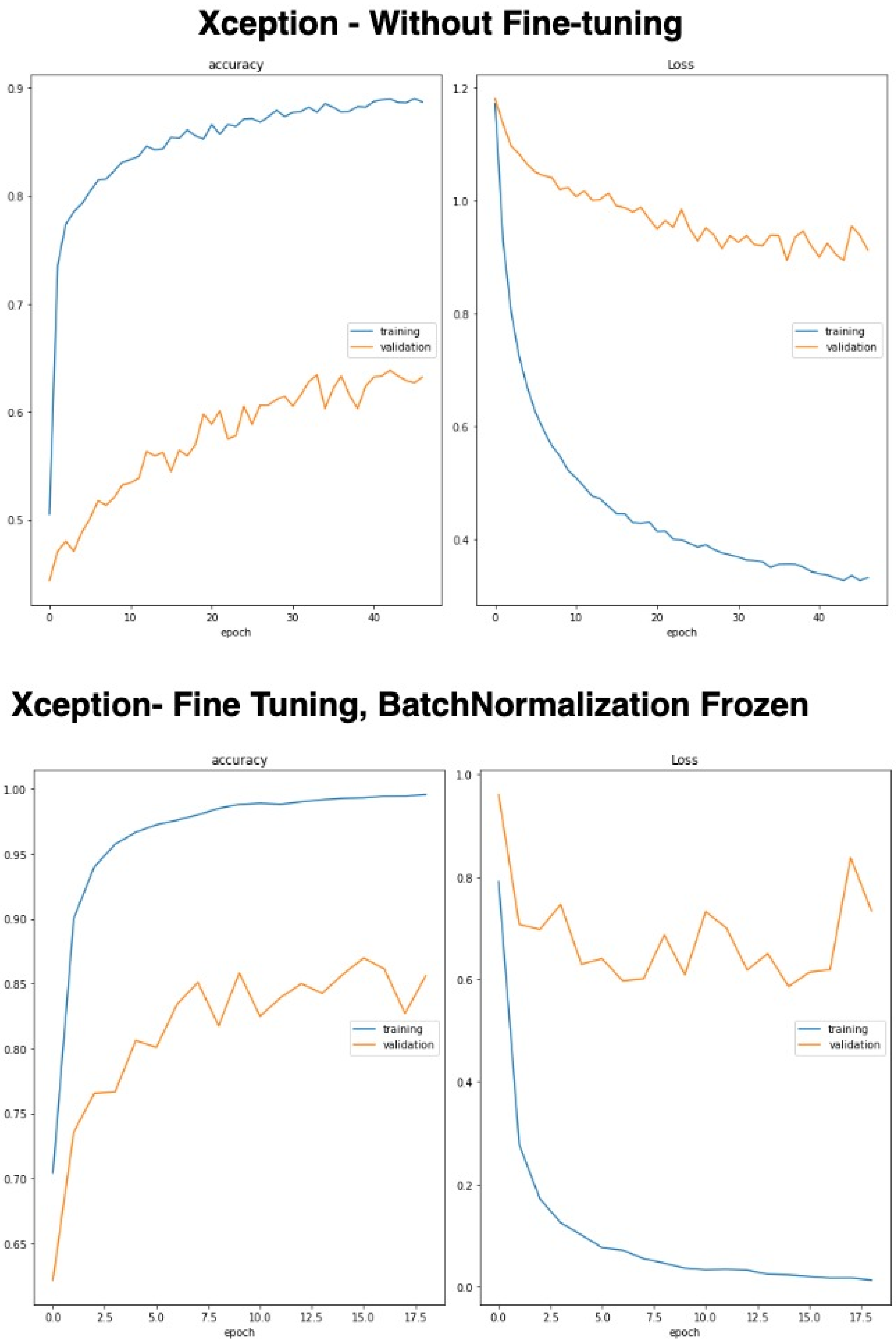

Figure S5. Training and validation curves (accuracy and loss) for Xception classifier

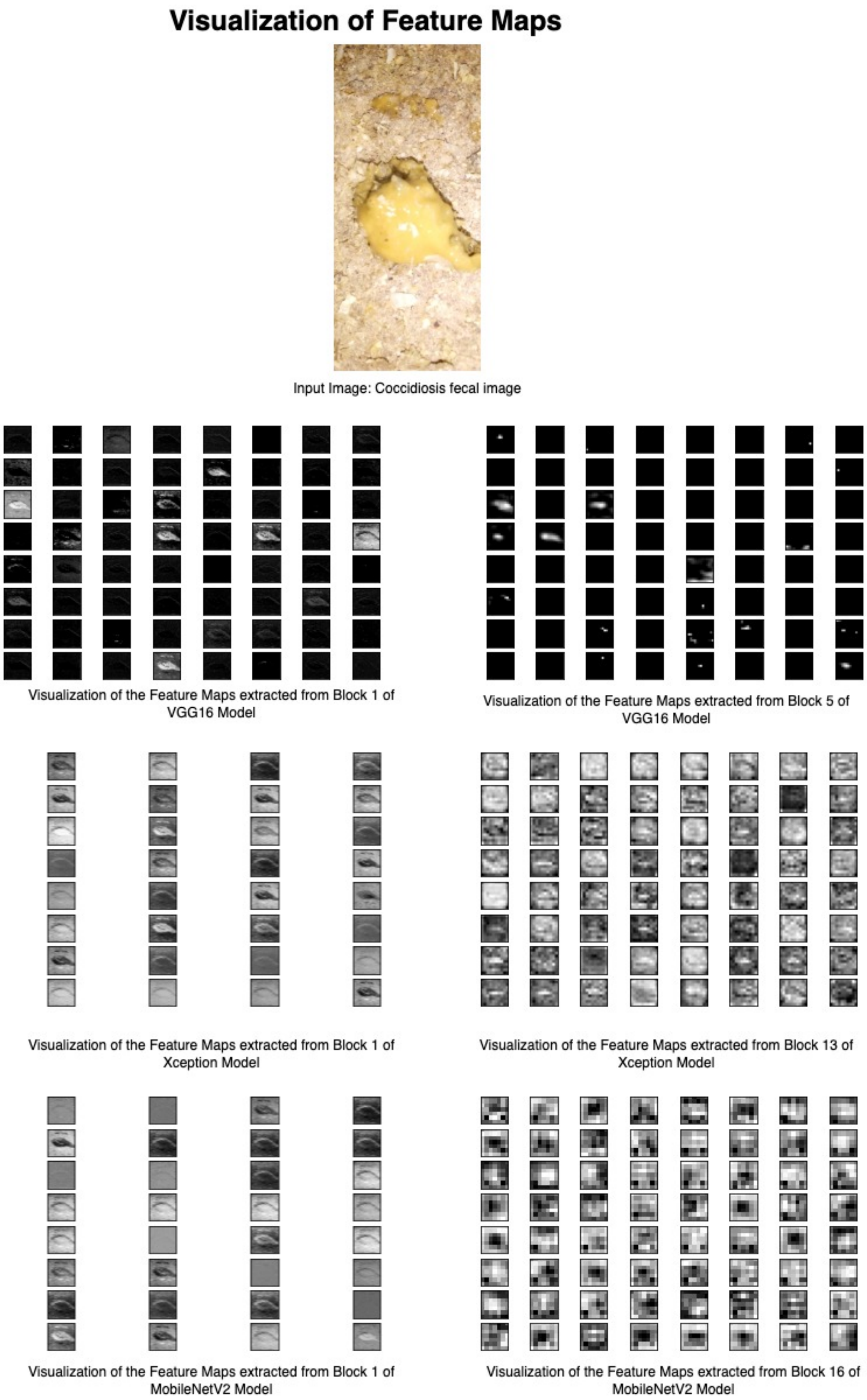

Figure S6. Visualization of Feature Maps of Coccidiosis input image

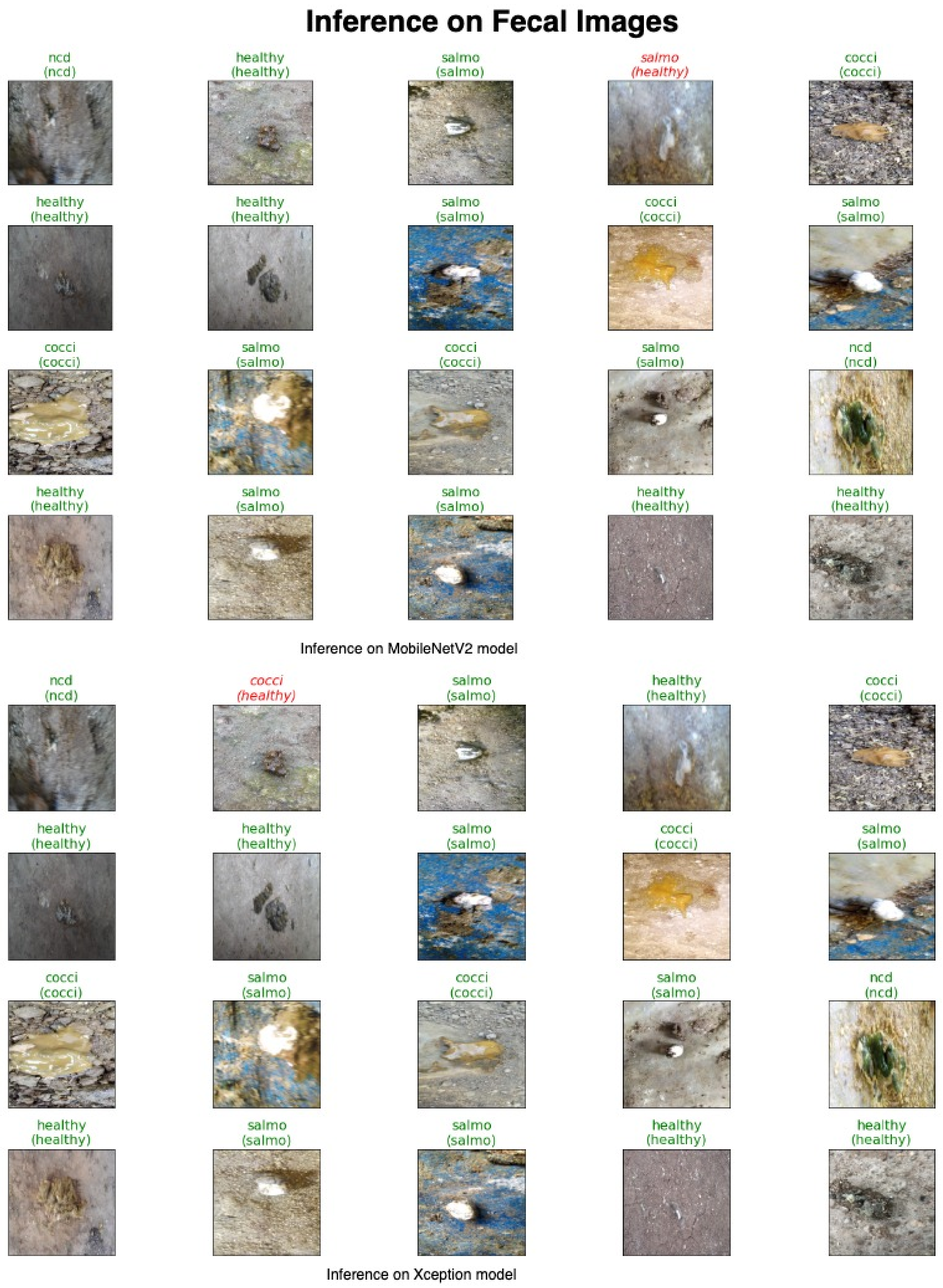

Figure S7. Inference on MobileNetV2 and Xception Models (green means correctly classified and red is classified incorrectly; the reference label is provided in parentheses below the classified label, and italicized when classified incorrectly)
